# Major ethnic group differences in breast cancer screening uptake in Scotland are not extinguished by adjustment for indices of geographical residence, area deprivation, long-term illness and education

**DOI:** 10.1038/bjc.2012.83

**Published:** 2012-03-13

**Authors:** N Bansal, R S Bhopal, M F C Steiner, D H Brewster

**Affiliations:** 1 Edinburgh Ethnicity Health Research Group, Centre for Population Health Sciences, University of Edinburgh, Teviot Place, Edinburgh EH8 9AG, UK; 2 Information Services Division, NHS National Services Scotland, Gyle Square, 1 South Gyle Crescent, Edinburgh EH12 9EB, UK

**Keywords:** breast cancer, cancer screening, ethnicity, inequalities

## Abstract

**Background:**

Breast cancer screening data generally show lower uptake in minority ethnic groups. We investigated whether such variations occur in Scotland.

**Methods:**

Using non-disclosive computerised linkage we combined Scottish breast screening and Census 2001 data. Non-attendance at first breast-screening invitation (2002–2008) was compared between 11 ethnic groups using age-adjusted risk ratios (RR) with 95% confidence intervals (CI), multiplied by 100, using Poisson regression.

**Results:**

Compared with the White Scottish (RR=100), non-attendance was similar for Other White British (99.5, 95% CI 96.1–103.2) and Chinese (112.8, 95% CI 96.3–132.2) and higher for Pakistani (181.7, 95% CI 164.9–200.2), African (162.2, 95% CI 130.8–201.1), Other South Asian (151.7, 95% CI 118.9–193.7) and Indian (141.7, 95% CI 121.1–165.7) groups. Adjustment for rural *vs* urban residence, long-term illness, area deprivation and education, associated with risk of non-attendance, increased the RR for non-attendance except for Pakistani women where it was modestly attenuated (RR=164.9, 149.4–182.1).

**Conclusion:**

Our data show important inequality in breast cancer screening uptake, not attenuated by potential confounding factors. Ethnic inequalities in breast screening attendance are of concern especially given evidence that the traditionally lower breast cancer rates in South Asian groups are converging towards the risks in the White UK population. Notwithstanding the forthcoming review of breast cancer screening, these data call for urgent action.

Migrant studies have demonstrated marked variation in cancer mortality by country of birth in Europe, with, for example, higher risks in Scottish-born and Irish-born and lower risk in South Asia-born migrants in England (Wild *et al*, 2006). Country of birth data together with limited studies on ethnicity assessed directly (Winter *et al*, 1999; Smith *et al*, 2003; Jack *et al*, 2007, 2009; Ali *et al*, 2010; Day *et al*, 2010), and on ethnic minority populations born in the country (Cummins *et al*, 2001; McKinney *et al*, 2003), show that such variations are mediated by environmental factors (Harding and Rosato, 1999). Changing risk with duration of residence (Harding, 2003) and convergence of risk factors, together with poorer declines in traditionally low-risk groups (Harding *et al*, 2009; Arnold *et al*, 2010; Mangtani *et al*, 2010), threaten the healthy migrant advantage (Zaman and Mangtani, 2007). Breast cancer is one of the most common cancers in women of all ethnic groups (Bhopal and Rankin, 1996) in the United Kingdom, United States and probably globally (Kamangar *et al*, 2006). Emerging evidence shows that rates of breast cancer in traditionally low-risk groups such as South Asian women are converging towards the majority White population in the United Kingdom (Winter *et al*, 1999; Jack *et al*, 2009; Ali *et al*, 2010) and the United States (Blesch *et al*, 1999).

Inequalities in health service use and treatment can augment ethnic differences in disease outcomes. For many years, there has been controversy about the balance of harms (such as over-diagnosis and over-treatment) and benefits of breast cancer screening (Bewley, 2011), leading to the recent establishment of an independent review in the United Kingdom (Richards, 2011). However, it is widely accepted that breast cancer screening can reduce the risk of disease-specific mortality through early detection and treatment, and pending the results of the review, women in the UK have been advised to continue to take up invitations for screening. In Scotland, since 1988 the United Kingdom National Health Service Breast Screening Programme (NHSBSP) has invited women aged 50 (to 64 until 2003–2004 and then to 70 years thereafter) for screening once every 3 years. However, the associated data set does not include ethnicity to allow assessment of inequalities in service use.

Data from England and the US suggest ethnic inequalities in cancer health service use. For example, studies have shown relatively advanced breast cancer stage of disease at diagnosis in Indian, Pakistani and Black women in the United Kingdom and United States (Li *et al*, 2003; Morris *et al*, 2007; Jack *et al*, 2009); diagnostic delay in Black and South Asians (Neal and Allgar, 2005); and ethnic inequalities in treatment (Jack *et al*, 2009) with poorer survival in Black African women in the UK (Jack *et al*, 2009). There is less information on screening uptake and inequalities in cancer service use in other minority ethnic groups such as the Chinese and Mixed Background groups and in non-majority White groups, which are particularly important given the heterogeneity in cancer risk noted in United Kingdom White groups (Harding *et al*, 2009). The non-White population is smaller in Scotland than in England, at the 2001 census constituting 2% of the total population (clustered mostly in Glasgow and Edinburgh) compared with England where it is 8%. Pakistanis form the majority (31.3%) of the non-White population, followed by Chinese (16%), Indians (14.8%), Mixed Background (12.6%) with Caribbean (1.8%) and Black Scottish (1.1%) forming the smallest groups. This is in contrast to England where Indians form the largest ethnic minority group and Chinese the smallest (General Register Office for Scotland, 2001).

The presence and extent of ethnic inequalities, and their relation to socio-economic factors, in Scotland are unknown. This information gap is contrary to the legal and policy commitments in Scotland, to improve the health of minority ethnic groups (Scottish Executive Health Department, 2002), reduce health inequalities (Scottish Executive, 1999) and promote racial equality (Home Office, 2001). These mandates were profoundly undermined by the lack of a sound evidence base to underpin policy development, service planning, delivery of health services and research (Netto *et al*, 2001).

To fill the information gap, the Scottish Health and Ethnicity Linkage Study was established (Bhopal *et al*, 2011). We investigated ethnic variations in breast screening uptake and tried to understand these in relation to socio-economic area deprivation, education, geography and health status.

## Methods

### Breast cancer screening data

Women invited for screening are selected and identified using the Community Health Index (CHI), which is linked to the Scottish Breast Screening Programme (SBSP) Information System. The SBSP Information System extracts details of eligible women for screening and these details are sent to the relevant GPs for checking; any discrepancies/amendments are then fed back and the system is updated. The SBSP Information System provides the SBSP with the functionality to invite eligible women via a call and recall facility, to allocate appointments and record the results of screening episodes. Once a woman has been selected for screening, a record is created for that individual and held on the SBSP Information System. Information relating to each step as a woman moves through her screening episode (including attendance) is collected on paper forms and saved on to the SBSP Information System. These data are collected for each individual for each individual episode. Patients only receive a reminder letter if they ‘Do Not Attend’ their appointment (regardless of their appointment type). Those due to attend the static centre are sent a reminder 4 weeks after their date of appointment and those due to attend a mobile unit are sent a reminder 2 weeks after their date of appointment. Information entered into the SBSP system is validated at the point of entry, which ensures data are accurate and of a high standard.

### Community Health Index to census linkage

As described previously (Fischbacher *et al*, 2007) (Bhopal *et al*, 2011), an anonymised data set containing census (respondent defined ethnicity and sociodemographic variables from the 2001 census) and various health outcomes, including breast screening uptake, was created using the probability linkage method, linking the census 2001 for Scotland to the Scottish CHI (register of patients using the NHS). Approximately 95% of the population enumerated in the 2001 census was linked to the CHI overall (4.65 million, with 85% or more linked in every ethnic group). Taking into account the census enumeration rate (96%), this represents about 91% of the population resident in Scotland in April 2001 (Bhopal *et al*, 2011).

### Data analysis

**Figure 1:**
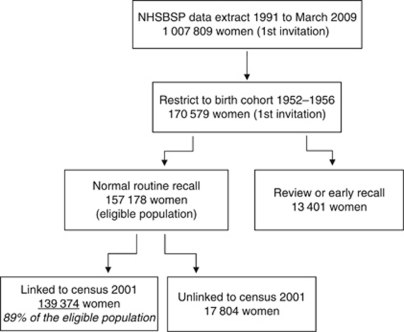
Selection of cohort.

As shown in Figure 1, we identified a cohort of women born between 1952 and 1956 and expected to have received a first routine breast screening invitation between 2002 and 2008 (allowing for the 3-year time period (50–53 years of age) over which a women can expect to receive first invitation). We limited the cohort to normal routine invitations only and excluded women being recalled early or under review for clinical reasons. Eighty-nine percent of breast screening records were linked to the census. We compared breast screening uptake by ethnic group. We identified potential confounders relating to socio-economic area deprivation (Scottish Index of Multiple Deprivation (SIMD) quintiles), education (here defined as having any recognised qualification *vs* none), geography (defined here as rural, small towns, and urban) and health status (long-term illness, yes/no) from the census data set and explored the association between these factors, breast screening uptake, and ethnic group.

Breast screening uptake (non-attendance) was compared between ethnic groups and data are presented here as age-adjusted risk ratios (RR) for non-attendance, with 95% confidence intervals around summary measures using Poisson's regression with robust variance. The standard comparison population was the White Scottish population at the 2001 census. Data were analysed using SAS version 9 (SAS Institute Inc., Cary, NC, USA).

Owing to small numbers, the following ethnic groups – African, Caribbean and Black Scottish or Other Black – have been amalgamated and are here referred to as the ‘African’ group; the ‘Other South Asians’ includes the Bangladeshi group.

In the results we focus on findings where the 95% CI does not include 100, the value for the standard White Scottish comparison population.

### Ethics and disclosure control

The analysis was conducted on a standalone computer in a locked room in the General Register Office for Scotland (Now National Records of Scotland) by named researchers (NB, MS) following a strict protocol to prevent inadvertent disclosure.

The work was approved by the Multicentre Research Ethics Committee (for Scotland) and the Privacy Advisory Committee of NHS National Services Scotland. The ethical and other permissions and related issues have been reported in detail, including an independent assessment by an ethicist (Boyd, 2007).

## Results

### Breast screening non-attendance

In all, 139 374 records were identified as being first routine invites between 2002 and 2008 from the NHSBSP data. Overall, 23% of the invited cohort did not attend (DNA), with clear ethnic variations (Table 1). Differences that are statistically robust are considered below. Table 2 shows that compared with the White Scottish (100), age-adjusted RR for non-attendance were higher for every ethnic group except the Other White British, Chinese and Other Ethnic groups, where they were similar. Risks of non-attendance were highest for Pakistani (181.7) followed by African (162.2), Other South Asian (151.7), Indian (141.7), Any Mixed Background (138.4) and Other White (125.3) women.

### Other factors associated with breast screening uptake

**Figure 2:**
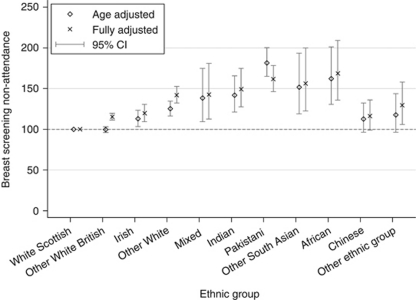
Non-attendance age- and fully-adjusted (age, education, deprivation, long-term illness and urban/rural status) RR with 95% confidence intervals by ethnic group.

Besides ethnicity, living in a rural *vs* urban area, having a long-term illness, area deprivation and individual educational status were factors associated with risk of non-attendance. Table 2 shows the effect of additional adjustment for each of these variables. Further adjustment for SIMD increased the RR in all ethnic groups relative to the White Scottish, incurring the largest change in most ethnic groups compared with other indicators. Age- and SIMD-adjusted ratios were now also higher for the Other White British, Chinese and Other Ethnic groups compared with the White Scottish. Adjustment for education increased RR in the White groups and decreased the RR in Pakistani, Indian and Chinese women. For Pakistani women, adjustment for education had the greatest attenuating effect reducing excess risk by 20%. Further adjustment for long-term illness and for urban/rural status incurred smaller changes compared with SIMD and education. Adjustment for all these factors, as shown in Figure 2, increased the RR in all non-Scottish groups except for Pakistani women where it was attenuated (164.9, 149.4 to 182.1).

## Discussion

### Principal findings

These first-ever Scottish data demonstrate that 23 years after the introduction of the United Kingdom national breast screening programme, ethnic inequalities in uptake at first invite are substantial for almost every non-white group in Scotland. These data are of concern especially given that women who attend breast screening at first invitation are more likely to attend for subsequent screens (Price *et al*, 2010). There are no published comparative ethnicity data to show how poor screening uptake is reflected in stage of disease at diagnosis in Scotland. However, drawing from English data, inequalities are likely to be considerable, and ethnic inequity in the extent of preventable cancer mortality may be marked, especially for Pakistani and African women.

### Findings in relation to the literature

Ethnic variations in use of cancer screening services are seen internationally (Kagawa-Singer and Pourat, 2000; Kandula *et al*, 2006; Ho and Dinh, 2011). Lower cancer screening uptake has been noted in migrant (Webb *et al*, 2004) and ethnic minority women in England with lower uptake of breast screening in South Asian (Szczepura *et al*, 2008; Price *et al*, 2010) and Black (Renshaw *et al*, 2010) women. Despite limitations of studies using other indicators as a proxy for ethnicity such as name identification (Cummins *et al*, 1999; Nanchahal *et al*, 2001) and area-based methods, these English data are compatible with our findings showing lower breast cancer screening in South Asian and African women. Our data show clear heterogeneity among South Asian women, with worse uptake for Pakistani women, followed by Other South Asian (mostly Bangladeshi) and with Indian women doing better than South Asian counterparts. This is consistent with previous data from England showing particularly low rates for Muslim South Asian women (Szczepura *et al*, 2008; Price *et al*, 2010). Surprisingly, we found that Chinese women had more similar uptake to White Scottish women, and not the poor utilisation characteristic of ethnic minority women and as seen in American Chinese women (Tu *et al*, 1999). Our study also demonstrated differences between White groups, with poorer uptake in Irish and Other White women but not in Other White British compared with the White Scottish. We found no other data exploring breast screening uptake in United Kingdom White groups.

Understanding variation between the White groups, and between ethnic minority women, particularly between the somewhat heterogeneous South Asian group and the Chinese, is potentially a key to understanding variation in service use.

Although socio-economic factors such as socio-economic deprivation (Power *et al*, 2009; Steele *et al*, 2010) and wealth (Moser *et al*, 2009) are important for service use, their role in explaining ethnic variations in breast screening uptake in the UK have not been fully studied. In our study, despite ethnic differences in area deprivation, education, long-term illness and urban/rural residence, these factors did little to attenuate ethnic differences in uptake between White Scottish and other White groups and between ethnic minority groups.

Studies exploring ethnic variations in breast screening uptake in the United Kingdom and United States suggest a strong role for cultural factors. Most of the data from England focus on South Asian women with data on Black and Chinese women originating mainly from the US. These data suggest that barriers to uptake include lack of knowledge and awareness of breast cancer and breast screening, and differences in perceived risk (Naish *et al*, 1994; Kernohan, 1996; Sadler *et al*, 2001, 2007; Watts *et al*, 2004; Jacobs *et al*, 2005; Thomas *et al*, 2005; Abdullahi *et al*, 2009; Robb *et al*, 2010). These, and other factors such as modesty, fear, embarrassment relating to breast examination particularly by male staff, differing attitudes, perceptions and beliefs of health and health service, language difficulties – both English proficiency and literacy in own language (Naish *et al*, 1994; Kernohan, 1996; Sadler *et al*, 2000; Jacobs *et al*, 2005; Thomas *et al*, 2005; Lee-Lin *et al*, 2007; Abdullahi *et al*, 2009; Banning, 2011), have been highlighted as explanatory factors for ethnic variations in uptake. For South Asian women in particular, low priority for self-care due to extended family responsibilities has been identified as an additional limiting factor (George and Ramkissoon, 1998).

Szczepura (2005) categorised these identified explanations for poor health care access into (1) ‘personal (intrinsic)’ factors (cultural differences, language and literacy and user ignorance) relating to the specific and particular needs of ethnic minority populations and (2) ‘organisational (extrinsic)’ factors (differential needs and provision, location of services and staff training needs). Both need to be considered when planning services for multi-ethnic and multicultural populations with the awareness that these needs may change (Szczepura, 2005) with level of acculturation, age and generational status of these communities.

There is a dearth of evidence for effective interventions to improve access in ethnic minority populations (Sokal, 2010). Given the complexity of cultural factors affecting uptake, evaluations of carefully tailored interventions are needed. A study in Canada showed the effectiveness of a culturally tailored and language-specific, written health education for South Asian women resulting in increased uptake of clinical breast examination (Ahmad *et al*, 2005). In London, a multi-pronged approach using targeted outreach work via community organisations and GP incentives to increase uptake, text message reminders and improved breast screening services, including a dedicated call-centre staff training, and interpreters, were seen to be important modifications in improving uptake (Eilbert *et al*, 2009).

Our data are of special concern given data showing advanced cancer stage at diagnosis in ethnic minority women (Cuthbertson *et al*, 2009), and are particularly worrying for South Asian women who are reported to present later for breast cancer and with larger primary tumours (Velikova *et al*, 2004). Data in Scotland on disease stage at presentation need to be examined by ethnic group.

### Strengths and limitations of the study

The strength of the study is the overall size, its population base, the availability of an ethnic code completed by the householder on behalf of the household, information on a wide range of ethnic groups studied simultaneously, information on relevant potential confounding factors, and being the first presentation of Scottish data by ethnic group. Recording of ethnicity data in the 2001 census is fairly complete (96%). The weaknesses of the study include the small population size for some non-White populations (African, Other South Asians, Any Mixed Background) requiring aggregation of heterogeneous groups such as African and Caribbean; and that 11% of breast screening records did not have a corresponding census file and we were therefore unable to explore the unlinked population. As our cohort is restricted to women attending breast screening in 2002 and beyond, it is possible that many of these (11%) were not resident in Scotland during the 2001 census. We do not have information on non-linking records and reasons for non-links may relate partly to failure to find (i.e., participant not registered with a GP or no contact with the NHS in Scotland) or match (misspelling or missing information on names or other identifiers used for matching) records.

## Conclusion

Our data showing substantial ethnic inequalities in breast screening attendance are of concern, especially given evidence suggesting that the traditionally lower breast cancer rates in South Asian groups are converging with higher risk White populations in the United Kingdom (Harding *et al*, 2009). Our data extend and corroborate data from England showing poorer uptake in all South Asian women, but with clear heterogeneity, not explained here by socioeconomic factors. Notwithstanding the forthcoming review of breast cancer screening (Bewley, 2011; Richards, 2011), these data call for urgent action. More work is needed to understand and address the low service use in these ethnic minority groups with particular urgency for Pakistani and African women. Meanwhile, policymakers and health-care staff need to respond to the existing evidence base highlighting multiple barriers affecting screening uptake in ethnic minority groups and ensure that services and staff are culturally sensitive and competent.

## Figures and Tables

**Table 1:** Number of women invited and percentage that did not attend (DNA) after first invitation for breast screening by ethnic group in Scotland (2002-2008)

**Ethnic group**	**DNA (*n*)**	**Invited (*n*)**	**DNA (%)**
White Scottish	28 398	123 026	23.1
Other White British	2541	11 056	23
White Irish	368	1411	26.1
Other White	542	1874	28.9
Indian	106	324	32.7
Pakistani	241	575	41.9
Other South Asian	42	120	35
Chinese	114	438	26
African	52	139	37.4
Any Mixed Background	48	150	32
Other Ethnic Group	71	261	27.2
Total	32 523	139 374	23.3

**Table 2:** Risk ratios for non-attendance adjusted by age, deprivation, education, long-term illness and urban/rural status with 95% CI by ethnic group

**Ethnic group**	**Age**	**Age and SIMD^a^**	**Age and education^b^**	**Age and long-term illness^c^**	**Age and urban/rural residence^d^**
White Scottish	100	100	100	100	100
					
*Other White British*	99.5 (96.1, 103.2)	110.9 (107.1, 115.0)	107.0 (103.2, 110.9)	101.3 (97.8, 105.0)	104.8 (101.1, 108.7)
White Irish	112.9 (103.3, 123.3)	118.4 (108.4, 129.4)	118.9 (108.8, 129.9)	113.6 (104.0, 124.1)	112.3 (102.8, 122.7)
Other White	125.3 (116.6, 134.6)	138.8 (129.2, 149.2)	135.2 (125.8, 145.3)	127.3 (118.5, 136.7)	127.3 (118.5, 136.8)
Indian	141.7 (121.1, 165.7)	158.8 (135.8, 185.6)	137.7 (117.6, 161.1)	139.7 (119.1, 163.8)	137.7 (117.7, 161.1)
Pakistani	181.7 (164.9, 200.2)	183.4 (166.3, 202.3)	162.3 (147.3, 178.9)	167.7 (151.9, 185.1)	173.4 (157.4, 191.1)
					
*Other South Asian*	151.7 (118.9, 193.7)	160.5 (125.5, 205.2)	156.0 (121.9, 199.5)	148.6 (116.5, 189.4)	147.8 (115.8, 188.7)
Chinese	112.8 (96.3, 132.2)	123.1 (105.0, 144.3)	103.1 (87.9, 120.9)	116.1 (99.1, 135.9)	109.6 (93.6, 128.5)
African	162.2 (130.8, 201.1)	163.9 (131.8, 203.8)	174.0 (140.2, 216.0)	162.0 (130.8, 200.7)	158.7 (128.2, 196.5)
					
Any Mixed Background	138.4 (109.5, 174.9)	142.7 (112.9, 180.4)	144.1 (114.0, 182.2)	136.7 (107.9, 173.1)	138.8 (109.6, 175.7)
					
Other Ethnic Group	117.8 (96.6, 143.6)	128.8 (105.6, 157.1)	119.0 (97.6, 145.2)	121.7 (99.7, 148.5)	117.6 (96.3, 143.6)

a Scottish Index of Multiple Deprivation (SIMD) quintiles.

b Qualification *vs* none.

c Defined as yes/no.

d Defined as rural, small towns, urban.
